# Are malaria transmission-blocking vaccines acceptable to high burden communities? Results from a mixed methods study in Bo, Sierra Leone

**DOI:** 10.1186/s12936-021-03723-0

**Published:** 2021-04-13

**Authors:** Kaci D. McCoy, Caroline T. Weldon, Rashid Ansumana, Joseph M. Lamin, David A. Stenger, Sadie J. Ryan, Kevin Bardosh, Kathryn H. Jacobsen, Rhoel R. Dinglasan

**Affiliations:** 1 CDC Southeastern Center of Excellence in Vector Borne Diseases, Gainesville, FL USA; 2 Mercy Hospital Research Laboratory, Bo, Sierra Leone; 3 School of Community Health Sciences, Njala University, Bo, Sierra Leone; 4 Center for Bio/Molecular Science and Engineering, Naval Research Laboratory, Washington, DC USA; 5 Center for One Health Research, School of Public Health, University of Washington, Seattle, WA USA; 6 Department of Global and Community Health, College of Health and Human Services, George Mason University, Fairfax, VA USA; 7 Emerging Pathogens Institute, Department of Infectious Diseases and Immunology, College of Veterinary Medicine, University of Florida, Gainesville, FL USA; 8 Department of Geography, College of Liberal Arts & Sciences, University of Florida, Gainesville, FL USA

**Keywords:** Malaria, Transmission-blocking vaccine (TBV), Acceptability, Bo, Sierra Leone

## Abstract

**Background:**

Malaria transmission-blocking vaccines (TBVs) could help break the cycle of malaria transmission by conferring *community* rather than *individual* protection. When introducing new intervention strategies, uptake is dependent on acceptability, not just efficacy. In this exploratory study on acceptability of TBVs in Sierra Leone, it was hypothesized that TBVs would be largely acceptable to adults and health workers in areas with relatively few ongoing malaria interventions, and that (i) knowledge of malaria and vaccines, (ii) health behaviours associated with malaria and vaccines, and (iii) attitudes towards different vaccines types could lead to greater TBV acceptability.

**Methods:**

This study used a mixed methods approach in Bo, Sierra Leone, to understand community knowledge, attitudes, and practices related to malaria and vaccination in general. This included: (i) a population-based cross-sectional survey (n=615 adults), (ii) 6 focus group discussions with parents, and (iii) 20 key informant interviews. The concept of a TBV was explained to participants before they were asked about their willingness to accept this vaccine modality as part of an integrated malaria elimination programme.

**Results:**

This study found that most adults would be willing to receive a TBV vaccine. Respondents noted mostly positive past experiences with adult and childhood vaccinations for other infectious diseases and high levels of engagement in other malaria prevention behaviors such as bed nets. Perceived barriers to TBV acceptance were largely focused on general community-level distribution of a vaccine, including personal fears of vaccination and possible costs. After an explanation of the TBV mechanism, nearly all focus group and interview participants believed that community members would accept the vaccine as part of an integrated malaria control approach. Both parents and health workers offered insight on how to successfully roll-out a future TBV vaccination programme.

**Conclusions:**

The willingness of community members in Bo, Sierra Leone to accept a TBV as part of an integrated anti-malarial strategy suggests that the atypical mechanism of TBV action might not be an obstacle to future clinical trials. This study’s findings suggests that perceived general barriers to vaccination implementation, such as perceived personal fears and vaccine cost, must be addressed in future clinical and implementation research studies.

**Supplementary Information:**

The online version contains supplementary material available at 10.1186/s12936-021-03723-0.

## Background

Malaria elimination and eradication remains one of the greatest global public health challenges. Approximately 405,000 malaria deaths still occur each year, with the greatest mortality rate among children who are under 5 years of age [1]. Malaria vaccines have been in the product development pipeline for over 50 years, with great hope but varying results due to the complex biology of the parasite. Phase 3 trials for the most advanced vaccine candidate (RTS,S/AS01 vaccine), which was given a positive scientific opinion in 2015 by the European Medicines Agency for the prevention of clinical malaria caused by *Plasmodium falciparum* in children, showed that four doses in children 5–17 years provided 36% protection over 4 years [2]. These modest results highlight the need for malaria elimination to be based on an integrated approach, aligned with local epidemiology, mosquito dynamics, and social and health system realities.

Given the current reality of pre-erythrocytic and blood stage vaccine efficacy, transmission-blocking vaccines (TBVs) have been identified as an intervention that can be paired with other malaria control efforts, including the RTS,S/AS01 vaccine, to work towards malaria elimination [3, 4]. TBVs could reduce malaria transmission by directly affecting obligatory development of *Plasmodium* parasites in *Anopheles* mosquito vectors [4–7]. TBVs have been developed as vaccine strategies for cytomegalovirus [8, 9] and human papillomavirus [10, 11]. While no TBVs have yet been approved for use against malaria, they are in development [12]. Currently, a malaria transmission-blocking vaccine (TBV) targeting Anopheline mosquito midgut-specific alanyl aminopeptidase N (AnAPN1), a highly conserved luminal midgut surface glycoprotein involved in blood meal digestion, has recently completed a process development study in anticipation for subsequent manufacture for a first-in-human clinical trial [13–15]. The AnAPN1 TBV is effective across malaria parasite and mosquito species and was designed to be co-administered with pre-erythrocytic vaccines, such as the RTS, S vaccine, in support of malaria elimination. It is also protein-based and designed to be easy to manufacture, facilitating global access via production by smaller, local pharmaceutical companies [4, 13]. At least six additional TBV candidates are currently in pre-clinical and clinical trials. These TBVs work similarly to inhibit stages of parasite development within the mosquito, but they target sexual stages of *Plasmodium* rather than the mosquito midgut, as with AnAPN1 [12].

**Fig. 1:**
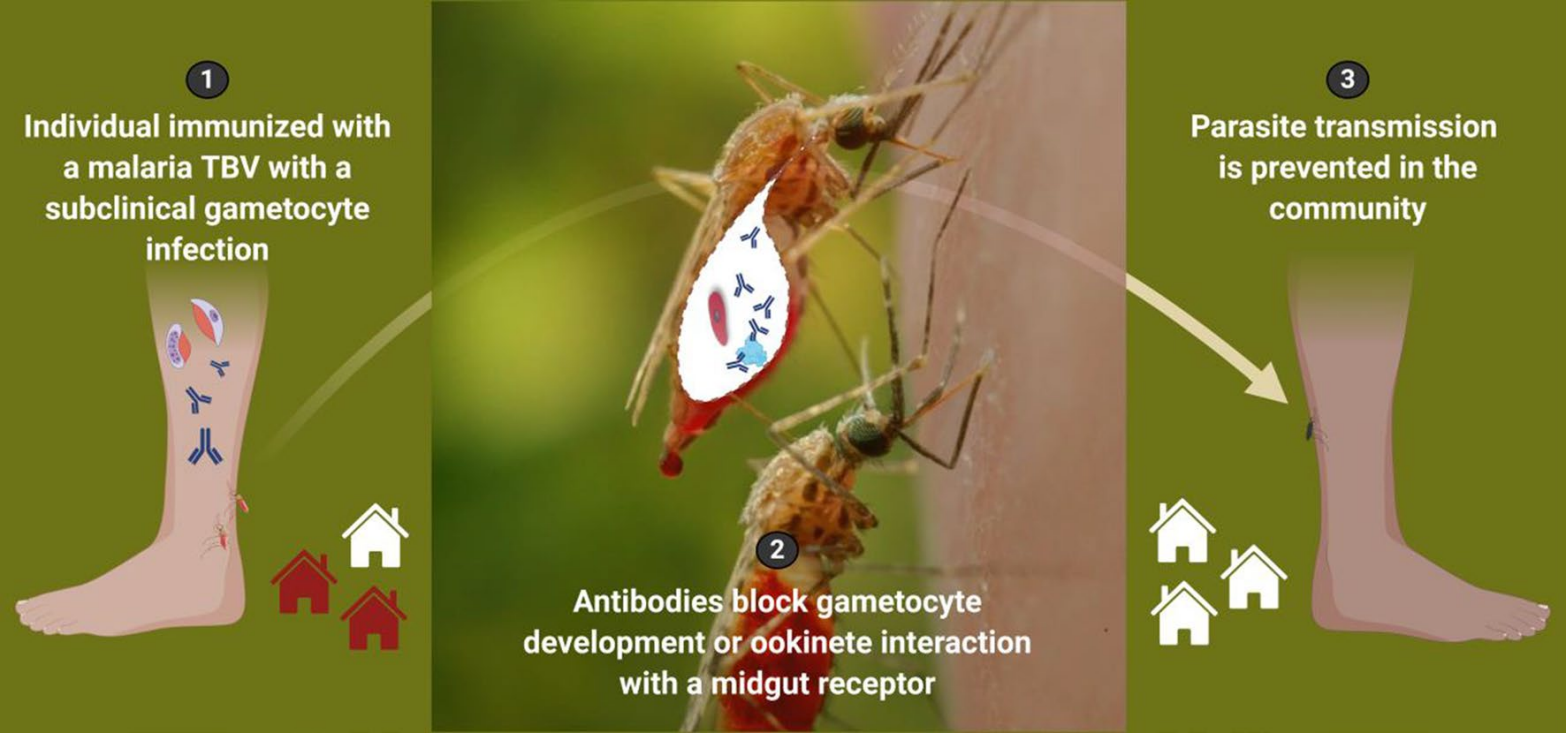
Malaria transmission-blocking vaccine concept for the prevention of community spread of malaria parasites.** a** Humans who receive and immunologically respond to a malaria transmission-blocking vaccine (TBV) develop antibodies that co-circulate with *Plasmodium* sexual stage gametocytes (*P. falciparum* gametocytes are shown in purple/red) during symptomatic and subclinical malaria infections. **b** *Anopheles* mosquitoes that blood feed on the immunized and infected host will pick up both the antibodies and the parasites into the mosquito midgut (red mosquito). Inside the midgut (inset, outline), the male and female gametocytes transform to gametes, then fertilize to form a motile zygote, called an ookinete. Antibodies elicited by TBVs that are taken up along with the parasite can either bind directly to the gametocyte (P230 or P48/45 [18–22]) or ookinete (P25 [23–25]) or to a critical mosquito receptor (AnAPN1 [13–15]) that the ookinete uses to enter the midgut cell to ensure sporogonic development continues. **c** By completely blocking or significantly reducing mosquito infections, TBVs prevent the cascade of secondary infections in a community when the mosquito takes another bloodmeal (uninfected, black mosquito). Homes in the community with infectious individuals are shown in dark red and homes without malaria infections are indicated in white. Middle image was adapted from Public Health Image Library

TBVs elicit antibodies against target surface antigens on the parasite gametocyte or ookinete stages, or mosquito midgut surface receptors that mediate ookinete attachment and subsequent establishment of the sporogonic cycle in the vector. These antibodies are taken up along with parasitized red blood cells (gametocyte sexual stages) into the mosquito during blood feeding on the immunized human host (Fig. 1). Immediately following uptake into the midgut, *Plasmodium* gametocytes undergo gametogenesis to form male and female gametes, which then fertilize to form the motile ookinete stage. Ookinetes exit the blood meal bolus, invade and traverse the midgut epithelium to form an oocyst, initiating sporogony. Transmission-blocking antibodies prevent either ookinete formation from gametocytes or ookinete invasion of the midgut, thereby preventing the cascade of secondary infections in humans when the mosquito takes another blood meal [4, 13, 16, 17].

Considerations for development of a TBV go well beyond biomedical research. As work moves from the bench into clinical trials, it is critical to explore the acceptability of this unique vaccine modality to at-risk communities; specifically, as TBVs confer protection at the community level and offer protection to the individual only through herd immunity, successful implementation of such a vaccine requires an understanding of the community perception of such an intervention [26, 27]. Only one published study has explored community acceptability of TBVs thus far, and that was conducted in Peru [28] not in sub-Saharan Africa, which is the region with the greatest malaria burden. Acceptability studies of non-TBV malaria vaccines in Kenya [29] and Togo [30] have found an openness to vaccinating infants and children against malaria. However, it has been also found that subsequently receiving a vaccine, once one is approved for widespread use, would depend on factors, such as attitudes of the community towards healthcare systems and vaccines, the perceived severity of malaria, and various cost considerations [31].

**Fig. 2:**
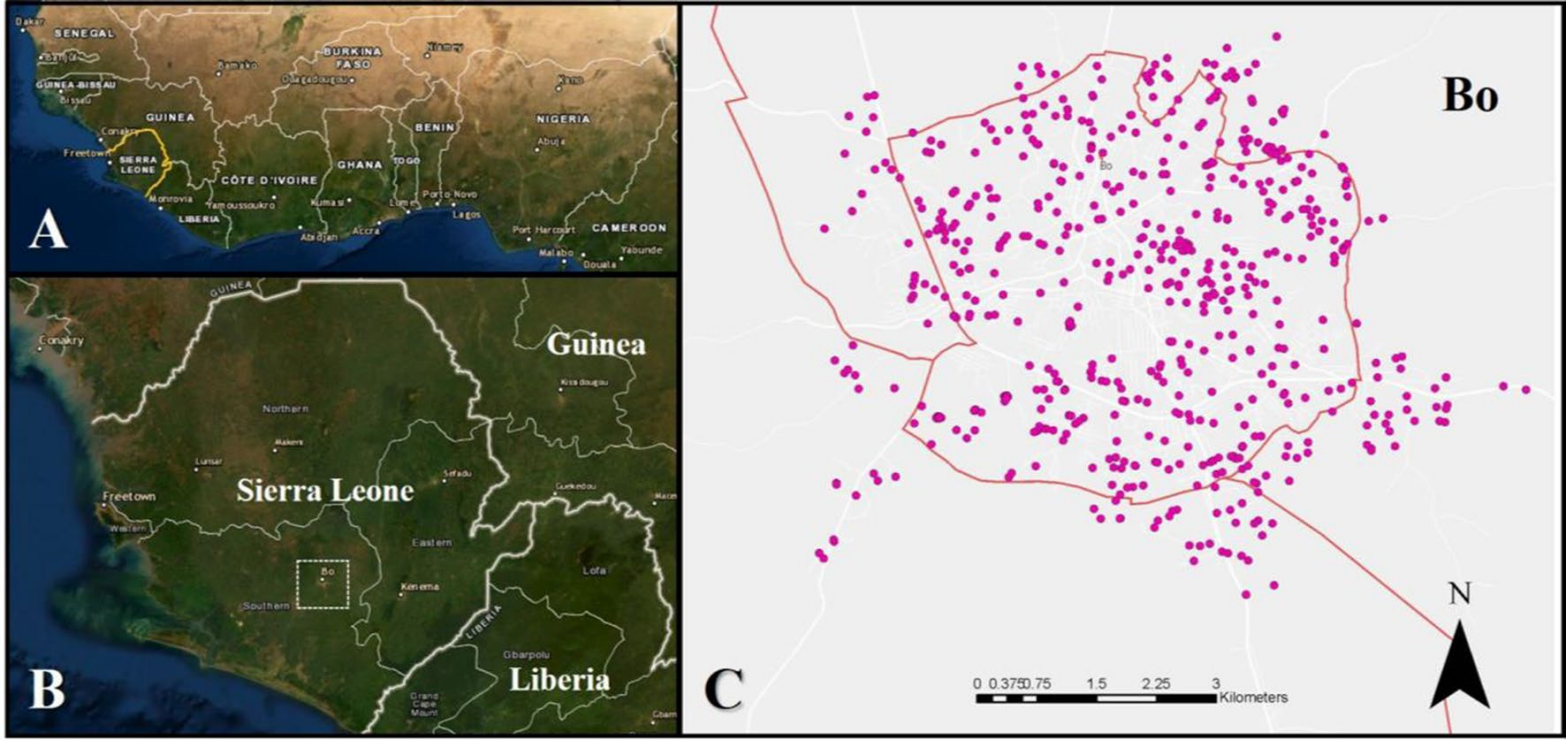
**a–c a** Sierra Leone (outlined in yellow) in context of West Africa; **b** Bo City (outlined in white) in relation to Sierra Leone and neighboring Guinea, Liberia; **c** Location of household surveys throughout Bo City, Sierra Leone

A mixed methods study was conducted in the city of Bo, Sierra Leone (Fig. 2) to better understand whether TBVs would be an acceptable type of malaria vaccine, as they act at the community level and within the mosquito population itself and hence do not confer direct, immediate benefit to the vaccinated individual. Knowledge, attitudes, and practices (KAP) related to (i) knowledge of malaria transmission, (ii) health behaviours associated with transmission, and (iii) attitudes towards different vaccines types, including TBVs were explored. It was hypothesized that knowledge of malaria transmission and engagement in malaria- and vaccine-related health behaviours would be associated with increased acceptability of a TBV. Promoters and barriers for a potential future TBV vaccine campaign were also identified to better understand community acceptability and implementation.

## Methods

### Study setting

Malaria remains the disease of highest burden in Sierra Leone [32], which has one of the highest prevalence rates of endemic *P. falciparum* malaria in the world [33] and high parasite prevalence (40% as of 2016) in children under five [34]. Compared to other countries in sub-Saharan Africa, Sierra Leone has not experienced frequent, large-scale malaria intervention or malaria vaccine studies within the last decade. While the National Malaria Control Programme conducted four rounds of insecticide-treated bed net (ITN) mass distribution campaigns, in 2006, 2010, 2014, and 2017, other interventions have been very limited throughout the past decade, including pilot testing of indoor residual spraying (IRS) in 2010–2012 in four districts; entomological monitoring in eight sentinel sites in 2018 (with a pilot test in 2010); and anti-malarial mass drug administration in select chiefdoms during the 2014–2015 Ebola Virus Disease outbreak [35]. The Global Fund and, as of 2017, the United States President’s Malaria Initiative, have been prominent international partners. At the time of the study, PMI-planned scale-ups of intervention activities were either building or had not yet been implemented, including social and behavioral change communication (SBCC) messaging training and IRS, respectively [35]. Due to the limited and intermittent nature of these past interventions, Sierra Leone can be reasonably considered relatively “malaria intervention-naïve” in the context of this study. Its communities are therefore prime settings to assess acceptability of TBVs, which are unique modalities in the repertoire of malaria vaccine candidates that have been developed over the past 50 years.

Bo District is one of the largest of 16 districts in the Southern Province of Sierra Leone (Fig. 2b), with a population of approximately 575,500, divided into 68 mutually exclusive neighborhoods or sections [36]. Bo District is culturally and ethnically diverse, although the Mende is the primary ethnic group [37]. Similar to the national average, 40% of children under five tested positive for malaria in Bo District in the 2016 Malaria Indicator Survey [34]. Bo Town, where this study was conducted, is the largest urban center in Bo District, with a population of 174,369 [36]. Bo Town’s infrastructure varies from the city center with busy paved roads to its less-built peri-urban outskirts. Bo is about 4 miles north-to-south and 4 miles east-to- west, with a radius of about 2 miles, giving it a small footprint with close proximity to very rural settings [38]. Like many other areas undergoing urbanization [39], malaria is recognized as a burden within Bo Town. For example, from February 2019 to 2021, over half of febrile patients who received testing at Mercy Hospital (n = 4137), a private hospital located within Bo Town that refers its patients to the Mercy Hospital Research Laboratory, were diagnosed with malaria [unpublished observations, Mercy Hospital]. Home to local universities and organizations with international and local reach, future TBV rollout sites are anticipated to have a similar environment to that of this study setting.

### Pilot testing

Pilot testing of quantitative and qualitative questions was conducted among enumerators and within the general population in Bo. This testing solicited enumerator feedback on question phrasing and allowed for preliminary screening of responses through the data collection platform. Trends in responses were examined for indications of misunderstandings or misrepresentations of questions. Following feedback, enumerators were given additional guidance on question phrasing and were retrained on the survey and interview tools.

### Household survey methods

A random location generator (http://www.geomidpoint.com/random/) was used to sample locations within Bo City. Based on the results of prior malaria surveys in Bo, it was estimated that about 500 participants would yield sufficient statistical power to examine the primary study questions. Six-hundred participants were recruited to ensure adequate power. To account for a greater population density near the city centre [40], a series of random samples was taken from five concentric circles centred on the city centre and with radii of 1 km, 2 km, 3 km, 4 km and 5 km [41]. Any locations that fell outside of the city limits or in uninhabited areas were removed from the sample.

Surveyors used the sample location coordinates to recruit a population-based sample for the quantitative survey. If the sampled location was residential, one adult from the residential structure was invited to participate in the study. If the nearest structure to the assigned coordinate was not residential, there were no eligible adults at a sampled residence, or the adults were not willing to participate at the residence, a “turn-to-the-right” approach was used, with the next residence to the right of the originally selected residence invited to participate. A local survey team from the Mercy Hospital Research Laboratory (MHRL) read the consent statement and answered any questions participants had about the study before beginning the interview. Consenting adults were asked to provide a signature, mark, or thumbprint on a copy of the informed consent statement as documentation of their willingness to participate in the research project. Surveys and consenting were conducted in English, Krio, or Mende, whichever language was preferred by the participant. The primary questionnaire consisted of 53 questions within six sections on demographics, malaria risk, malaria diagnosis and treatment, malaria prevention, vaccination, and wealth assessment; study participants with a child less than 10 years old were asked an additional 34 questions about one of their eligible children’s health and about malaria prevention, diagnosis, and treatment related to their child (see Additional file 1). The questionnaire was pilot tested within the community prior to data collection. Data collection was conducted using OpenDataKit (ODK) accessed on the surveyor’s mobile device, and daily collated survey reports were downloaded from ODK every night. Descriptive analyses were conducted using EpiInfo 7. This study was approved by the Institutional Review Board (IRB) of Njala University, University of Florida (#201901536), and George Mason University (#1410559).

The questionnaire is organized by five categories titled demographics, malaria risks, malaria prevention, vaccination, and wealth assessment. These segments are aimed to explore the (i) knowledge of malaria transmission, (ii) health behaviours associated with transmission, and (iii) attitudes towards different vaccines types. The vaccination portion of the questionnaire asked about previous vaccine uptake and attitudes regarding vaccine modalities and need for repeated doses. Participants were asked if they would want to be vaccinated if a safe and effective malaria vaccine was available for adults, their willingness to pay for a malaria vaccine, the amount they would be willing to pay, and the preferred number of required doses (one dose vs. annual doses). The questionnaire script then explained that “*A traditional vaccine would keep you from getting malaria from a mosquito*,” then asked, “*Would you be willing to get a vaccine that would prevent you from getting malaria from a mosquito?*” Next, the script explained that “*A different type of vaccine would keep people who already have malaria parasites in their blood from passing the parasites to mosquitoes that bite them. This would slow or stop transmission in a community if enough people were vaccinated*.” This was followed by the question “*Would you be willing to be vaccinated as part of a community malaria control programme?*”

### Focus group discussions (FGDs) and key informant interviews (KIIs)

Six FGDs comprising 7–15 mixed gender participants were conducted. Participants in the FGDs were selected to represent caregivers and adults with children in order to capture knowledge, attitudes, and practices related to malaria, malaria prevention, and vaccination of people who might be responsible for decisions about children’s health. FGDs lasted for 15–20 minutes and were conducted by three teams of three research assistants: two members facilitated the discussion, and the third member took notes. For the KIIs, interviewees represented both male and female key community health leaders, including government hospital staff, community healthcare workers, researchers, and public health officials; these interviews were conducted to gain understanding of healthcare workers’ perspectives on community knowledge, attitudes, and practices related to malaria and malaria prevention, as well as insights on vaccinations in the community. KIIs lasting approximately 20 minutes were conducted with community health leaders by research assistants. FGDs and KIIs were conducted in English, Krio, or Mende, whichever language was preferred by the participants. Both FGD and KII teams led discussions using a set of guidelines (see Additional file 2) and recorded the sessions on their mobile devices to later transcribe and translate from Krio or Mende (as necessary) to English. At the onset of the focus group and individual interviews, the research team obtained verbal consent. Once the research team reached what they considered to be a high level of saturation of key themes—the point where the same responses and information were emerging in each session [42, 43]—the FGDs and KIIs were concluded. This decision was also influenced by logistical and time constraints, and further research is recommended to clarify specific qualitative findings in more depth, as herein.

After two members of the survey team checked the transcriptions for accuracy, the FGD and KII audio recordings were deleted for participants’ privacy. Additionally, no names were included in the transcripts. The qualitative data were analysed manually using a coded thematic approach. A codebook was developed based on themes included in the qualitative discussion guideline and was reviewed by the research team. Data were compiled and divided by subthemes, which were further grouped by similar concepts and ideas. The main findings from each subtheme were then reviewed by two researchers and summarized and interpreted (see Additional file 3).

## Results

Data were collected in July 2019. Survey participant demographics are shown in Table 1. Qualitative results are described within the themed sub section below.

**Table 1:** Quantitative survey respondent demographics by frequency (%)

Gender		
Female	366	(59.5%)
Male	249	(40.5%)
Age		
18–30	315	(51.2%)
31–40	138	(22.4%)
41–50	67	(10.9%)
51–60	55	(8.9%)
61–70	33	(5.4%)
71+	7	(1.1%)
Marital status		
Coupled	389	(63.2%)
Single	226	(36.8%)
Household characteristics		
No children	131	(21.3%)
1+ child <10 years old	337	(54.8%)
Child/all children ≥ 10 years old	147	(23.9%)
Religion		
Islam	338	(55.0%)
Christianity	263	(42.8%)
None/no response	14	(2.3%)
Years of formal education		
0	153	(24.9%)
1–6	55	(8.9%)
7–9	84	(13.7%)
10–12	163	(26.5%)
13–16	154	(25.0%)
17+	6	(1.0%)

### Malaria knowledge and health behaviours

Malaria transmission knowledge, defined as knowledge that mosquitoes spread malaria, was very high in Bo city. Of the 615 questionnaire respondents, 59% (363) correctly identified “mosquito bite” as the most common route of malaria transmission, and an additional 30% (182) of respondents correctly identified this mode, as well as at least one other incorrect mode of transmission **(**Table 2). All focus groups and nearly all key informants consistently linked malaria transmission with mosquitoes; three focus group participants specifically noted it was the female *Anopheles* mosquito. Malaria vaccines were generally accepted among the participants, regardless of their transmission knowledge. Of participants who either did or did not identify “mosquito bite” as a mode of transmission, 97% and 94%, respectively, would be willing to accept a traditional malaria vaccine. Of these same groups, 96% and 91%, respectively, would be willing to accept a TBV. This indicates a high level of acceptability (>90%) of both types of vaccine, regardless of measured transmission knowledge.

**Table 2:** Acceptability of vaccine modalities by adult and child knowledge, attitudes, and practices related to malaria and vaccines

	Number (%) of respondents with characteristic	Number (%) of TBV acceptability with characteristic	Number (%) of TBV acceptability without characteristic	Number (%) of traditional vaccine acceptability with characteristic	Number (%) of traditional vaccine acceptability without characteristic
Adult knowledge and prevention (n = 615)					
Identified mosquito bite as a mode of malaria parasite transmission (includes correct mode only and those that also include 1+ incorrect modes)	545 (88.6)	521 (95.6)	64 (91.4)	526 (96.5)	66 (94.3)
* Only identified correct mode of malaria parasite transmission (i.e., mosquito bite)*	363 (59.0)	346 (95.3)	239 (94.8)	348 (95.9)	244 (96.8)
House has bed nets	497 (80.8)	469 (94.4)	116 (98.3)	475 (95.6)	117 (99.2)
Slept under bed net last night (n = 497)	401 (80.7)	378 (94.3)	91 (94.8)	382 (95.3)	93 (96.9)
Vaccinated against any diseases	484 (78.7)	463 (95.7)	83 (96.5)	468 (96.7)	83 (96.5)
Adult Vaccination Attitudes (n = 615)					
Vaccines are for both adults and children	471 (76.6)	453 (96.2)	–	459 (97.5)	–
* Vaccines are for children only*	93 (15.1)	87 (93.6)	–	87 (93.6)	–
Vaccines are safe	552 (89.8)	528 (95.7)	42 (93.3)	535 (96.9)	42 (93.3)
Trust the health staff who give vaccines	546 (88.8)	524 (96.0)	54 (90.0)	530 (97.1)	55 (91.7)
Would want to be vaccinated if safe and effective malaria vaccine available	588 (95.6)	575 (97.8)	7 (38.9)	583 (99.2)	3 (33.3)
Willing to pay for malaria vaccine	389 (63.3)	380 (97.7)	205 (90.7)	386 (99.2)	206 (91.2)
Willing to be vaccinated every year	573 (93.2)	562 (98.1)	18 (58.1)	569 (99.3)	18 (58.1)
Willing to get traditional malaria vaccine	592 (96.3)	583 (98.5)	2 (12.5)	–	–
Willing to get malaria TBV	585 (95.1)	–	–	583 (99.7)	6 (28.6)
Have concerns about vaccines for adults	461 (75.0)	444 (96.3)	141 (91.6)	451 (97.8)	141 (91.6)

	Number (%) of respondents who indicated child has characteristic	Number (%) of TBV acceptability with characteristic	Number (%) of TBV acceptability without characteristic	Number (%) of traditional vaccine acceptability with characteristic	Number (%) of traditional vaccine acceptability without characteristic
Child Prevention (n = 337)					
Slept under bed net last night	241 (71.5)	237 (98.3)	89 (100)	237 (98.3)	89 (100)
Vaccinated against any diseases	323 (95.9)	319 (98.8)	8 (100)	319 (98.8)	8 (100)
Child Vaccination Attitudes (n = 337)					
Would want child vaccinated if safe and effective malaria vaccine available	326 (96.7)	323 (99.1)	10 (90.9)	323 (99.1)	10 (90.9)
Willing to pay for child’s malaria vaccine	188 (55.8)	186 (98.9)	147 (98.7)	186 (98.9)	147 (98.7)
Willing to have child vaccinated every year	325 (96.4)	324 (99.7)	8 (72.7)	324 (99.7)	8 (72.7)
Willing to give child traditional malaria vaccine	333 (98.8)	333 (100)	0 (0)	–	–
Willing to give child malaria TBV	333 (98.8)	–	–	333 (100)	0 (0)
Have concerns about vaccines for children	261 (77.5)	257 (98.5)	76 (100)	257 (98.5)	76 (100)

Similarly, the acceptability of malaria vaccines was high regardless of previous health behaviours. Of participants who either reported being vaccinated against any diseases in the past (79%) or did not report prior vaccination, 97% and 97%, respectively, would be willing to accept a traditional malaria vaccine. Of these same groups, 96% and 97%, respectively, would be willing to accept a TBV. Among those who reported they did not know if they had previously been vaccinated, acceptance of a traditional malaria vaccine or a TBV were 91% and 87%, respectively. This indicates that the acceptability of either type of malaria vaccine was high, regardless of previous vaccination history, with only a slight impact of knowledge of prior vaccination history appearing associated with reduced acceptability.

Most respondents reported engaging in malaria prevention activities; 81% (497/615) reported owning a bed net, and 81% (401/497) of these individuals reported sleeping under a bed net the night before. Malaria vaccines were similarly acceptable among participants regardless of whether they slept under bed nets. Of respondents who did or did not sleep under a bed net the night before the survey, 95% and 97%, respectively, would be willing to accept a traditional malaria vaccine. Of these same groups, 94% and 95%, respectively, would be willing to accept a TBV.

### Vaccine attitudes and acceptability

The majority of survey respondents (79%) reported having been previously vaccinated against at least one disease (Table 2), and nearly all the survey respondents (96%) with a child under 10 years old reported that their children were vaccinated against at least one disease, with 90% (552) agreeing that “vaccines are safe” (Table 2). This attitude was also reflected in FGDs, where the majority of participants accepted vaccines to “*prevent you from acquiring disease*” and to “*improve immunity*.” Health workers reported that measles, polio, hepatitis B, tuberculosis, diphtheria, rubella, and yellow fever vaccines had previously been administered in the community.

When asked about a proposed malaria vaccine, 96% (588) of survey participants would want to be vaccinated if a safe and effective malaria vaccine was available. The acceptability was the same when participants were specifically asked about receiving a “traditional” malaria vaccine (i.e., a vaccine that prevents the recipient from acquiring the malaria parasite from an infected mosquito), with 96% (592) of survey questionnaire participants being willing to accept such a vaccine. Similarly, for participants with children under age 10, 97% (326) would want their child vaccinated if a safe and effective malaria vaccine was available. This general acceptability was echoed in the qualitative data, as the majority of focus groups and interviewees were willing to accept a malaria vaccine for the benefit of their own health, stating such things as: “*prevention is better than cure*” and “*health is wealth*.” The primary reason reported by these groups for accepting a malaria vaccine was to prevent themselves from malaria infection.

After the explanation of a transmission-blocking vaccine concept, 95% (585) of survey participants indicated willingness to receive this type of vaccine for malaria and 99% (333) of surveyed adults with children were willing for their children to receive this type of vaccine. Of the 4.9% (30) survey respondents who would not accept the vaccine or did not know if they would accept a proposed TBV, only 1.5% (9) said they would accept a traditional malaria vaccine. FGD and KII participants expressed interest in being “*a part of the malaria control programme*” and participating in this effort for their community because “*people are always sick with malaria*” and “*if there is a vaccine, it will lessen the burden.*” Although the most common reason for accepting the proposed TBV was to improve community health and reduce malaria cases, two focus group participants responded, “*because it prevents me from getting malaria, I will take it*” and “*of course, because I want to prevent myself from getting malaria*”, making it unclear if they understood the transmission-blocking vaccine mechanism.

### Community implementation

Personal fears were a primary reason for unwillingness to receive vaccines; identified concerns included fear of reactions such as hand swelling, slight fever, nervous system reactions, and general sickness after injection, as well as a general fear of needles. Mistrust in the government and unfounded rumors about vaccine campaigns causing infertility or spreading other infectious diseases were other reasons reported for avoidance of vaccination in the community. Although the community members reported general vaccine acceptance, 75% (461) of survey participants indicated having at least some concern about vaccines for adults and children. While 95% (585) of survey participants and 99% (333) of adults surveyed with children were willing to vaccinate themselves or their child with a TBV, focus group participants and key informants also stated that “*some people in our community will not take this vaccine*.” When asked for reasons why others might decline a TBV, they described the same types of fears and rumours about general vaccines that they expressed as personal hesitancies, including adverse effects, the idea that vaccinations could be harmful and/or meant to cause sickness, and fear of needles or injections.

When asked about willingness to pay for the TBV, only 63% (389) of surveyed adults and 56% (188) of the surveyed adults with children under the age of 10 expressed willingness to pay out-of-pocket for a vaccine for themselves or for their children, respectively. The mean prices participants would be willing to pay were 9,042 Sierra Leonean Leones (about $0.99 USD at the time of the study) for themselves and 6,252 Sierra Leonean Leones (about $0.69 USD at the time of the study) for their children. FGD participants highlighted the importance of the TBV vaccination being offered free of cost to recipients. Interviewees also warned that they had experienced supply shortages in past vaccine campaigns and stated that those supply chain management problems led to loss of community interest.

Key informants indicated that supplemental education and sensitization—focused outreach by which community healthcare workers introduce and share information with community members regarding health campaigns, interventions—will be key when introducing a new type of vaccine in the community and recommended coupling the TBV campaign with other existing vector control methods that are already in use to encourage participation. Previous vaccination campaigns have reportedly been successful in the community due to health education in local languages (i.e., Krio, Mende) and the use of social mobilization through the radio.

### Discussion and conclusions

This study is the first to assess the knowledge, attitudes, and practices related to TBV acceptability in sub-Saharan Africa. It was hypothesized that knowledge of malaria transmission and engagement in malaria- and vaccine-related health behaviours would be associated with increased acceptability of a TBV. However, the acceptability was high in participants regardless of their level of malaria transmission knowledge or engagement in malaria prevention activities. Participants generally showed high levels of knowledge of malaria transmission and most already participated in existing interventions, perhaps due to the recent PMI SBCC campaigns [35]. With rates of acceptability exceeding 90%, with the exception of participants who could not recall their vaccination history (87% acceptability), there was little (< 5%) difference in TBV acceptability between participants with complete or incomplete knowledge of malaria transmission, or who had or had not previously engaged in interventions.

Vaccine acceptability was high in general and for both types of proposed malaria vaccines (592 and 585 of 615 respondents), echoing previous findings that also found high vaccine acceptance rates for Ebola vaccines in Sierra Leone [44–46]. Importantly, there was no meaningful difference in acceptability between a proposed “traditional” malaria vaccine and a TBV. The most frequently mentioned reason for accepting the proposed TBV was positively contributing to community health, and that attitude indicated an understanding of the TBV paradigm. This was also the case for a community attitudes study toward MDA for control and elimination of neglected tropical diseases in a nearby county in Liberia, bordering Sierra Leone to the east [47]. The community leaders surveyed emphasized the communal benefits and duty to contribute to the betterment of their town, similar to the value of community health in Bo.

While Bo is described as relatively malaria-intervention naïve, the use of vaccines to prevent other diseases was not new. The ubiquity of references to the “five vaccines for children under five” campaign, in addition to recent Ebola vaccination campaigns—which included community engagement and outreach to healthcare workers [44–46, 48]—imply pre-existing vaccine knowledge and awareness which may have led to an increased understanding and acceptability of vaccines. Previous exposure to vaccine campaigns influenced key informant responses about anticipated acceptability of malaria vaccines, as they reported prior success using sensitization and education campaigns to address fears and rumours about vaccines. These messaging strategies may be employed in subsequent TBV studies.

With high reported individual willingness to accept the vaccines, the main anticipated barriers were related to implementation rather than the TBV’s mode of action; no individuals indicated a lack of direct individual protection as a reason to not accept the vaccine. The main concerns raised in FGDs and KIIs were vaccine safety and vaccine cost, which were similar themes as those identified in studies of acceptability of other vaccines in Sierra Leone, including Ebola and influenza [44, 48, 49]. Cost, in particular, is anticipated to be the main barrier to widespread vaccine uptake after a malaria vaccine is licensed for use. While this was expected—Sierra Leone’s Free Health Care Initiative [50] provides free routine immunizations for children under the age of five, so caregivers do not expect to pay for vaccinations—this barrier reinforces the need for support by national and international stakeholders, who will likely need to cover the cost of such a vaccine programme. The logistical burdens could be reduced by integrating the TBV into an existing national or regional programme for both children and adults [27].

Fears about vaccine safety and preference for vector control methods can be addressed through sensitization campaigns, which were identified by participants as successful in previous vaccination campaigns. Other studies exploring community perceptions of malaria and vaccines in sub-Saharan Africa similarly found lack of vaccine information, including that on safety and efficacy, as constraints for implementation [51, 52]. Coupling specific messaging designed by local leaders that highlights the role of a TBV within a comprehensive malaria control programme could help alleviate these concerns and overcome potential acceptability issues.

### Limitations and lessons learned

One of the strengths of this study was the use of trained research assistants from MHRL and Njala University, which are located in Bo city and have been partnered on multiple prior health-related research projects. However, some respondents, including health staff, had prior interactions with the researchers, and that may have contributed to some social desirability bias. This extensive local knowledge of the local research team, however, also allowed for generation and implementation of a population-based random sample, as well as effective communication, translation, and transcription in local languages and English.

The messaging and descriptions of proposed malaria vaccines may have resulted in ineffective communications of the difference between a “traditional” vaccine and a TBV. Pilot testing of the quantitative survey and qualitative interview guide found a need to rephrase questions about “traditional” vaccines, as the use of the word “traditional” to describe a typical, individual-focused vaccine approach (i.e., non-TBV approach) often led to a lack of acceptance in the vaccine, as it was negatively interpreted as traditional medicine *(vs.* “Western” medicine). This community perception was also documented in Bo during the Ebola outbreak [53]. The final questionnaire and interview guide included lengthier descriptions of both types of vaccines, but the term may still have had a negative connotation. The pilot testing did not reveal issues of participants misunderstanding the modality of the TBV; however, it is possible that it may not have been described equivalently to all participants enrolled in the study throughout the entire study period. “Self-protection from malaria” was identified as an enabler to TBV acceptability in the FGDs, as some individuals in the qualitative arm of the study failed to correctly identify who would and would not be protected by a TBV. Communication about vaccine science will need to be improved in future studies of malaria vaccines to ensure clear understanding of the key differences between types of vaccinations. Placing greater emphasis on visual communication may enhance understanding and enable greater community engagement with participatory research, as described by Enria *et al.* [42] in an Ebola vaccine trial in Sierra Leone. Additional and repeated questioning directly assessing the participants’ understanding of the TBV modality should be considered for future studies.

The assumption that Bo was a relatively malaria intervention-naïve setting due to a short history of intermittent interventions may have overlooked the impact of the number of malaria interventions and vector investigations occurring in and around Bo around the time of the study, such as scaling-up of ITPp and IRS in 2018 and ITPi (intermittent preventive treatment of malaria during infancy) in 2019 [1, 35]. Exposure to these interventions may have factored into acceptability of the proposed TBV. While many future TBV rollout sites are anticipated to be similar to Bo in terms of infrastructure and demographics, lending the results useful to future trials, the generalizability of this study is currently limited to urban to peri-urban, malaria endemic areas with highly educated populations. Therefore, future studies should target varied settings to broaden the generalizability of these results and inform implementation efforts.

Several important issues were not directly addressed in this study and would benefit from future in-depth qualitative and/or focused ethnographic research. This includes identifying social groups that might be less likely or more likely to accept the TBV; improving the understanding of how different levels of immunological protection influence acceptability; clarifying how specific local linguistic and cultural terms may be (mis)understood in future health communication as related to TBVs; and exploring how the therapeutic value of TBVs may influence community risk behaviors and normative prevention practices for malaria. With these limitations and lessons learned from this rapid formative study in mind, a second phase of community acceptability testing prior to vaccine rollout, involving a human-centered design approach to developing a tool for exploring community perceptions and acceptability of a TBV is recommended: formative interviews, a prototype, piloting the prototype and finally adapting the prototype into a final product*.* Follow-up studies should include more in-depth questioning on ethnographic factors and how TBV modalities are understood, which could inform future community education work and clinical trials. As sensitization was identified as a main player in acceptability, this also provides an opportunity to guide and refine future messaging to incorporate educational materials and modality illustrations.

### Conclusions and implications

This study shows that most community members, child caregivers, and healthcare workers in Bo, Sierra Leone are willing to receive a TBV such as the AnAPN1 vaccine. The main drivers of acceptability may not be centred on knowledge and health behaviour, but instead anchored on implementation concerns such as cost and general apprehension of vaccinations. While the results of this study may be generalizable to other areas which experience endemic malaria and have a similar built environment, infrastructure, and socio-demographic structure, similar surveys may be conducted in other settings where implementation of a TBV may be under consideration. Future studies should consider exploring potential promoter factors, such as enhanced understanding of vaccine modalities and ethnographic factors in health communication. Such research would be beneficial for an effective future integration of a TBV into a broader campaign to reduce malaria transmission, *en route* to elimination.

## Supplementary Information

**Additional file 1.** Discussion guide.

**Additional file 2.** Questionnaire.

**Additional file 3.** Codebook.

## Data Availability

The datasets used and/or analysed during the current study are available from the corresponding author on reasonable request. The materials supporting data collection are included within the Additional files (see Additional file1–3).

## Abbreviations

TBV: Transmission blocking vaccine
FGDs: Focus group discussions
KI: Key informant
KIIs: Key informant interviews
MHRL: Mercy Hospital Research Laboratory
ODK: OpenDataKit
