# Supplementary material for: Are malaria transmission-blocking vaccines acceptable to high burden communities? Results from a mixed methods study in Bo, Sierra Leone
Source: Malar J. 2021 Apr 13;20:183. doi: 10.1186/s12936-021-03723-0 (PMC8045381; doi:10.1186/s12936-021-03723-0)
Supplement: Supplementary file 1 — Additional file 1. Discussion guide. [file 12936_2021_3723_MOESM1_ESM.pdf]

Focus group and semi-structured interview question guide

1. Are you concerned about malaria in your community?
2. How often are people sick with malaria?
3. Do people die from malaria here?
4. How do people in your community understand how malaria is transmitted/spread?
5. From your understanding, what diseases are protected against through vaccines?
6. Does everyone accept vaccines here in this community?
7. Do some people refuse vaccines? Why?
8. Can adults receive vaccines or only children?
9. Have you heard about vaccines for Ebola? What do you think about it? What do people say about it?
10. A traditional vaccine would keep you from getting malaria from a mosquito. Would you be willing to get this type of vaccine?
11. A different type of vaccine would keep people who already have malaria parasites in their blood from passing the parasites to mosquitoes that bite them. This would slow or stop the spread of disease in a community, if enough people were vaccinated. Would you be willing to be vaccinated as part of a community malaria control program?
12. What community concerns, resistances or questions would you anticipate arising if this sort of vaccine was offered in your community?
