# Supplementary material for: Are malaria transmission-blocking vaccines acceptable to high burden communities? Results from a mixed methods study in Bo, Sierra Leone
Source: Malar J. 2021 Apr 13;20:183. doi: 10.1186/s12936-021-03723-0 (PMC8045381; doi:10.1186/s12936-021-03723-0)
Supplement: Supplementary file 2 — Additional file 2. Questionnaire. [file 12936_2021_3723_MOESM2_ESM.pdf]

Attitudes and practices related to malaria vaccine acceptability in Bo, Sierra Leone

Questionnaire

Survey #	
Interviewer	
Neighborhood	

Getting Started (*to be filled out by the interviewer*)

Has the consent form been completed?	☐ Yes
What is the gender of the study participant?	☐ Female<br>☐ Male

Demographics

1	**What is your age in years?**	*Enter a whole number, like 19 or 75.*	☐ ___________
	Is the individual age 18 or older?	*If the age in years is less than 18, the participant is <u>not</u> eligible for the study.*	☐ Yes
2	**Are you currently married (or partnered), or are you single?**	*Check only one box.*	☐ Married / partnered<br>☐ Single / divorced / separated / widowed
3	**Do you have any children (of any ages, including adult children)?**	*If the answer to this question is YES, then ask the follow-up question about the age of the children.*	☐ Yes<br>☐ No
3a	*<u>If YES</u>,* **do you have any children who are less than 10 years old?**	*If the answer to this question is YES, then the child questions will be asked at the end of the interview.*	☐ Yes<br>☐ No
4	**How many years of formal education did you complete?**	*Enter a whole number, like 19 or 75.*	☐ ___________
5	**What is your religion, if any?**	*Check only one box.*	☐ Christian<br>☐ Muslim<br>☐ Christian and Muslim<br>☐ None<br>☐ Other: _____

			☐ Prefer not to respond
6	**As of today, how would you rate your general health?**	*Check only one box.*	☐ Excellent<br>☐ Good<br>☐ Fair<br>☐ Poor<br>☐ Don't know / no answer
7	**Would you say that your health is better, worse, or about the same as most people your age?**	*Check only one box.*	☐ Better<br>☐ Worse<br>☐ About the same<br>☐ Don't know / no answer

Malaria Risk

8	**Have you ever had malaria in your lifetime?**		☐ Yes<br>☐ No
9	**Have you had malaria in the past year?**		☐ Yes<br>☐ No
10	**How would you rate your likelihood of becoming ill with malaria during the next 12 months: very high, high, moderate, low, or very low?**	*Check only one box.*	☐ Very high<br>☐ High<br>☐ Moderate<br>☐ Low<br>☐ Very low
11	**Do you feel that your risk of malaria is present all of the time, most of the time, some of the time, rarely, or never during the year?**	*Check only one box.*	☐ All of the time<br>☐ Most of the time<br>☐ Some of the time<br>☐ Rarely<br>☐ Never

Malaria Diagnosis and Treatment

12	**Have you had a fever at any time in the past month?**	*If the answer to this question is YES, then ask ALL of the follow-up questions about testing and diagnosis. If no, then skip to the next section.*	☐ Yes<br>☐ No

12a	***If YES*, did you go to a hospital or clinic because of your fever?**		☐ Yes<br>☐ No
12b	**Did you consult with a doctor, nurse, pharmacist, or other health worker about your fever?**		☐ Yes<br>☐ No
12c	**Did you have your blood tested at a laboratory during your illness?**		☐ Yes<br>☐ No
12d	**Did you take any medications, pills, herbs, or other treatments for your fever?**	*If yes, then ask the follow up question about types of treatments.*	☐ Yes<br>☐ No
12d2	***If YES*, what did you take?**	*Check or list ALL treatments mentioned. Many individuals will have taken 5 or more different treatments. All should be listed here.*	☐ Paracetamol<br>☐ ASAQ (artesunate-amodiaquine)<br>☐ moringa<br>☐ ________________<br>☐ ________________<br>☐ ________________<br>☐ ________________<br>☐ ________________

Malaria Prevention

13	**What is the most common way for malaria to be transmitted**	*Do NOT read the list. Just check the 1 answer provided by the participant as the MOST common.*	☐ Mosquito bite<br>☐ Close contact with someone who has malaria<br>☐ Dirty water<br>☐ I don't know<br>☐ Others (*write replies*): ____________________
14	**Which types of people have given you helpful information about how to prevent malaria?**	*Check or list ALL items mentioned, but do NOT read the list out loud.*	☐ Doctors / nurses / health workers<br>☐ Malaria control teams<br>☐ Family members<br>☐ Neighbors<br>☐ Friends<br>☐ Teachers<br>☐ Religious leaders

			☐ Others (*write replies*):<br>____________________
15	**What media sources (if any) have given you helpful information about how to prevent malaria?**		☐ Radio<br>☐ Television<br>☐ Newspapers<br>☐ Internet<br>☐ Billboards / outdoor banners<br>☐ Others (*write replies*):<br>____________________
16	**What actions do you take to prevent yourself from getting malaria?**	*Check or list ALL items mentioned, but do NOT read the list out loud.*	☐ Mosquito nets<br>☐ Close doors and windows<br>☐ Screens on doors and windows<br>☐ Insecticides in or around home<br>☐ Mosquito coils<br>☐ Covering clothing<br>☐ Others (*write replies*):<br>____________________
17	**Does your household have any mosquito nets?**	*If yes, then ask the 2 follow up questions about bednet use.*	☐ Yes<br>☐ No
17a	*If YES*, **how many mosquito nets does your household have?**	*Enter a whole number*	☐ __________
17b	**Did you sleep under a mosquito net last night?**		☐ Yes<br>☐ No

Vaccination

18	**Have you ever been vaccinated against any diseases (recently or in the past)?**	*Ask the follow up questions even if the answer to this question is 'NO'.*	☐ Yes<br>☐ No<br>☐ I don't know
19a	**Have you ever been vaccinated against<br>… measles?**		☐ Yes<br>☐ No<br>☐ I don't know
19b	**… polio?**		☐ Yes<br>☐ No<br>☐ I don't know
19c	**… tetanus?**		☐ Yes<br>☐ No

			☐ I don’t know
19d	**… yellow fever?**		☐ Yes<br>☐ No<br>☐ I don’t know
19e	**… hepatitis A virus?**		☐ Yes<br>☐ No<br>☐ I don’t know
19f	**… malaria?**		☐ Yes<br>☐ No<br>☐ I don’t know
19g	**… Ebola?**		☐ Yes<br>☐ No<br>☐ I don’t know
20	**In the past 5 years, has a doctor, nurse, public health worker, or someone else recommended that you receive a vaccine?**	*If yes, then ask the follow up question about the vaccine.*	☐ Yes<br>☐ No<br>☐ I don’t remember
20a	*If YES*, **did you receive that vaccine?**	*If no, then ask the follow up question about not getting the vaccine.*	☐ Yes<br>☐ No<br>☐ I don’t remember
20b	*If NO*, **why not?**	*Check or list ALL items mentioned, but do NOT read the list out loud. You may need to write several responses on the lines at the end of the list.*	☐ I did not think the vaccine was needed<br>☐ I use other methods to protect myself from the disease<br>☐ I did not think the vaccine would be safe<br>☐ I was worried about side effects<br>☐ I did not think the vaccine would be effective<br>☐ I thought the vaccine would cost too much<br>☐ I did not know where to get the vaccine<br>☐ I heard or read bad things about the vaccine<br>☐ I don’t like needles<br>☐ I was too busy / I could not leave work or home to get the vaccine

			☐ I did not know where to get reliable information about the vaccine<br>☐ I don't remember<br>☐ Others (*write replies*): ____________________
21	**Do you think that vaccines are just for children or are for both children and adults**	*Check only one box.*	☐ Only children<br>☐ Only adults<br>☐ Both children and adults<br>☐ I don't know / No opinion
22	**Do you believe vaccines are safe?**		☐ Yes<br>☐ No<br>☐ Other: ____________
23	**Do you trust the health staff who give out vaccines in your area?**		☐ Yes<br>☐ No<br>☐ Other: _____________
23	**If a safe and effective malaria vaccine was available for adults, would you want to be vaccinated**		☐ Yes<br>☐ No<br>☐ I don't know
24	**Would you be willing to pay to receive a malaria vaccine?**	*If yes, ask the follow-up question about price.*	☐ Yes<br>☐ No
24a	*If YES*, **how much would you be willing to pay?**	*Enter a number.*	☐ __________
25	**Would you be willing to receive a malaria vaccine if you had to be vaccinated every year rather than just one time?**		☐ Yes<br>☐ No<br>☐ I don't know
26	**A traditional vaccine would keep you from getting malaria from a mosquito. Would you be willing to get a vaccine that would prevent you from getting malaria from a mosquito?**		☐ Yes<br>☐ No<br>☐ I don't know
27	**A different type of vaccine would keep people who already have malaria parasites in their blood from passing the parasites to mosquitoes that bite them. This would slow or stop transmission in a community, if enough people were vaccinated. Would you be willing to be vaccinated**		☐ Yes<br>☐ No<br>☐ I don't know

	**as part of a community malaria control program?**		
28	**Do you have any concerns about vaccines for adults?**	*If yes, ask the follow-up question about price.*	☐ Yes<br>☐ No
28a	*If YES*, **what are your concerns?**	*List ALL items mentioned. You may need to write several responses on the lines.*	☐ ________________<br>☐ ________________<br>☐ ________________
			☐

Wealth Assessment

Is there electricity in the home?	☐ Yes<br>☐ No<br>☐ I don't know
What is the flooring material in the home?	☐ Earth, sand<br>☐ Dung<br>☐ Wood/planks<br>☐ Palm/bamboo<br>☐ Vinyl or asphalt strips<br>☐ Ceramic tiles<br>☐ Cement<br>☐ Carpet<br>☐ Other<br>☐ No response
How many rooms in the home are used for sleeping?	☐ One<br>☐ Two<br>☐ Three or more<br>☐ No response
Where does the household cook food?	☐ In the house<br>☐ In a separate building<br>☐ Outdoors<br>☐ No food cooked in household<br>☐ Other<br>☐ No response
What type of cooking fuel is used?	☐ Electricity<br>☐ LPH/natural gas/biogas<br>☐ Kerosene

	☐ Coal/lignite<br>☐ Charcoal<br>☐ Wood<br>☐ Straw/shrubs/grass<br>☐ Agricultural crop<br>☐ Other<br>☐ No food cooked in household<br>☐ No response

FOR PARTICIPANTS WHO HAVE AT LEAST ONE CHILD LESS THAN 10 YEARS OLD…

Child Eligibility

0a	**How many children do you have who are less than 10 years old?**	*Enter a whole number.*	☐ ____
0b	**How many of these children are less than 5 years old?**		☐ ____
0c	**How many of the children are 5 to 9 years old?**		☐ ____
		Confirm that the sum of children <5 and 5-9 is the total number of children <10.	☐ The sum is correct.

Choose only 1 child to answer questions about…

If the participant has more than 1 child under 10 years of age, choose 1 child to answer these questions about using this priority list:

- *If only one child is ages 5 to 9, use that child for the survey.*
- *If all the children are less than 5 years old, choose the oldest under-5 child.*
- *If more than one child is ages 5 to 9, select just one to answer the survey about.*

Child Demographics

1	**What is the age of the child you are going to answer the questions about?**	*Enter a whole number.*	☐ ____
2	**Is this child a boy or a girl?**		☐ Boy<br>☐ Girl
3	**Does this child attend school?**		☐ Yes<br>☐ No

Malaria Diagnosis and Treatment

4	**Has this child had a fever at any time in the past month?**	*If the answer to this question is YES, then ask ALL of the follow-up questions about testing and diagnosis. If no, then skip to the next section.*	☐ Yes<br>☐ No
4b	*If YES,* **did you take the child to a hospital or clinic because of the fever?**		☐ Yes<br>☐ No
4c	**Did you consult with a doctor, nurse, pharmacist, or other**		☐ Yes<br>☐ No

	**health worker about your child's fever?**		
4d	**Did you have your child's blood tested at a laboratory during your illness?**		☐ Yes<br>☐ No
4e	**Did you give your child any medications, pills, herbs, or other treatments for your fever?**	*If yes, then ask the follow up question about types of treatments.*	☐ Yes<br>☐ No
4e2	*If YES*, **what did you give your child?**	*Check or list ALL treatments mentioned. Many individuals will have taken 5 or more different treatments. All should be listed here.*	☐ Paracetamol<br>☐ ASAQ (artesunate-amodiaquine)<br>☐ moringa<br>☐ ________________<br>☐ ________________<br>☐ ________________<br>☐ ________________<br>☐ ________________

Child Disease Prevention

5	**Did your child sleep under a mosquito net last night?**		☐ Yes<br>☐ No<br>☐ I don't know
6	**Has your child ever been vaccinated against any diseases?**	*Ask the follow up questions even if the answer to this question is 'NO'.*	☐ Yes<br>☐ No<br>☐ I don't know
7a	**Has your child ever been vaccinated against:<br>… measles?**		☐ Yes<br>☐ No<br>☐ I don't know
7b	**… polio?**		☐ Yes<br>☐ No<br>☐ I don't know
7c	**… hepatitis B virus?**		☐ Yes<br>☐ No<br>☐ I don't know
7d	**… tetanus?**		☐ Yes<br>☐ No<br>☐ I don't know
7e	**… yellow fever?**		☐ Yes

			☐ No<br>☐ I don't know
7f	**… pneumococcus?**		☐ Yes<br>☐ No<br>☐ I don't know
7g	**… rotavirus?**		☐ Yes<br>☐ No<br>☐ I don't know
7h	**… malaria?**		☐ Yes<br>☐ No<br>☐ I don't know
7i	**… Zika?**		☐ Yes<br>☐ No<br>☐ I don't know
8	**In the past 5 years, has a doctor, nurse, public health worker, or someone else recommended that your child receive a vaccine?**	*If yes, then ask the follow up question about the vaccine.*	☐ Yes<br>☐ No<br>☐ I don't remember
18b	*<u>If YES</u>,* **did your child receive that vaccine?**	*If no, then ask the follow up question about not getting the vaccine.*	☐ Yes<br>☐ No<br>☐ I don't remember
18c	*<u>If NO</u>,* **why not?**	*Check or list <u>ALL</u> items mentioned, but do NOT read the list out loud. You may need to write several responses on the lines at the end of the list.*	☐ I did not think the vaccine was needed<br>☐ I use other methods to protect my child from the disease<br>☐ I did not think the vaccine would be safe<br>☐ I was worried about side effects<br>☐ I did not think the vaccine would be effective<br>☐ I thought the vaccine would cost too much<br>☐ I did not know where to get the vaccine<br>☐ I heard or read bad things about the vaccine<br>☐ My child does not like needles

			☐ I was too busy / I could not leave work or home to get my child vaccinated<br>☐ I did not know where to get reliable information about the vaccine<br>☐ I don't remember<br>☐ Others (*write replies*): ____________________
19	**If a safe and effective malaria vaccine was available for children, would you want to be vaccinated**		☐ Yes<br>☐ No<br>☐ I don't know
20	**Would you be willing to pay for your child to receive a malaria vaccine?**	*If yes, ask the follow-up question about price.*	☐ Yes<br>☐ No
20b	*If YES*, **how much would you be willing to pay?**	*Enter a number.*	☐ __________
21	**Would you be willing for your child to receive a malaria vaccine if he/she had to be vaccinated every year rather than just one time?**		☐ Yes<br>☐ No<br>☐ I don't know
22	**A traditional vaccine would keep your child from getting malaria from a mosquito. Would you be willing for your child to get a vaccine that would prevent your child from getting malaria from a mosquito?**		☐ Yes<br>☐ No<br>☐ I don't know
23	**A different type of vaccine would keep people who already have malaria parasites in their blood from passing the parasites to mosquitoes that bite them. This would slow or stop transmission in a community, if enough people were vaccinated. Would you be willing for your child to be vaccinated as part of a community malaria control program?**		☐ Yes<br>☐ No<br>☐ I don't know

24	**Do you have any concerns about vaccines for children?**	*If yes, ask the follow-up question about concerns.*	☐ Yes<br>☐ No
24b	*If YES*, **what are your concerns?**	*List ALL items mentioned. You may need to write several responses on the lines.*	☐ ________________<br>☐ ________________<br>☐ ________________
