# Supplementary material for: Are malaria transmission-blocking vaccines acceptable to high burden communities? Results from a mixed methods study in Bo, Sierra Leone
Source: Malar J. 2021 Apr 13;20:183. doi: 10.1186/s12936-021-03723-0 (PMC8045381; doi:10.1186/s12936-021-03723-0)
Supplement: Supplementary file 3 — Additional file 3. Codebook. [file 12936_2021_3723_MOESM3_ESM.pdf]

Theme	Sub-theme/code	Text
Malaria in the community	Severity	Interview 1: “They don’t go up to severe case most of the malaria cases that are coming here are uncomplicated malaria, they don’t die of it.”<br><br>Interview 2: “Yes, I am concerned about malaria because it is a deadly disease that is why I am very concerned about it as a health professional.”<br><br>“because they take it as a simple thing – ‘is just malaria’ they just buy paracetomol or some anti-malaria drug and just take it. They don’t know the gravity of the sickness, so people die almost every day of malaria.”<br><br>Interview 3: “With regards the concern, malaria is common and prevalent in Bo town generally. Everywhere you go, you see malaria patients and most of our patients that we deal with here are malaria related patients. “<br><br>[Do people die from malaria here?] “No, no, no. We have not got that kind of case yet. That is why when we see the severity of case, we because we do day care we don’t admit here that’s our problem.”
	Frequency	Interview 1: “Of course, I have concern because there are a lot of malaria cases coming up from that particular community. “<br><br>“Regularly, when I check them they do positive [for malaria].”<br><br>Interview 2: “Very often, mostly all the patients coming to the hospital are most always positive with malaria parasite. Out of hundred percent, ninety five percent which are always positive with malaria.”<br><br>Interview 3: “With regards the concern, malaria is common and prevalent in Bo town generally. Everywhere you go, you see malaria patients and most of our patients that we deal with here are malaria related patients. “

		"Almost out of every ten patients, you get like eight patients that have malaria."
Population at risk	Only children at risk for malaria	
	Adults at risk for malaria	
Symptoms	Symptoms/experiences	
Transmission knowledge	Correct knowledge of person-person transmission	Interview 2: "When you are talking about the community and the people as a whole, some people are aware of malaria that mosquito transfers malaria from one person to another infected mosquito."
	Correct knowledge of mosquito transmission	Interview 1: "they know malaria is transmitted by mosquito."<br>"they will say through mosquito bite and most of them know that"<br>Interview 2: "When you are talking about the community and the people as a whole, some people are aware of malaria that mosquito transfers malaria from one person to another infected mosquito."<br>"But malaria is transmitted from a mosquito, and it always have somewhere to breed around the community, if the community is not clean."<br>Interview 3: "A lot of people know how malaria is transmitted. A lot of people they know malaria is transmitted through mosquito bite, especially mosquito that carrying the malaria parasite itself people know that"
	Knowledge gaps in malaria transmission	Interview 2: "Then some will tell you that, if you eat too much of palm oil, I will get malaria. Some will tell you that if I suck oranges, I will get malaria."<br>"Sometimes I even laugh because people it like they need to create awareness in the people again about malaria, the spread of malaria at times we have to talk to them, give health talks, that malaria is not transmitted by foods, like that we have measured."

Risk factors	Environment or<br><br>hygiene	Interview 1: "environment is not so clean and that one encourages mosquitoes"<br><br>"But malaria is transmitted from a mosquito, and it always have somewhere to breed around the community, if the community is not clean."<br><br>Interview 2: "….if you do not have the preventative method because of their community is very filthy they have breeding site for mosquitoes…."
	Mosquito breeding sites	Interview 2: "But malaria is transmitted from a mosquito, and it always have somewhere to breed around the community, if the community is not clean."
	Adherence to treatment	Interview 1: "when they come we give them the malaria treatment, maybe they are not taking it correctly"<br><br>"and I think those are things the environment, adherence to treatment that's all that responsible for seeing malaria patients are coming in on and off for every month"<br><br>"I had to explain to the mother that it should not be like that, every month the baby takes malaria treatment, I asked what is your problem about this child do you put this child under mosquito net at night or are you giving the treatment correctly"
Prevention	Prevention methods used	Interview 3: "We advise them to get mosquito bed or bed nets, we advise them not to stay out long at night and we advise them to always make sure they close their windows, as early as possible and their door so that they prevent malaria mosquito from coming in."
	Lack of prevention methods used	Interview 2: "They do not sleep under mosquito net, no repellent,"

Treatment	Treatment methods	Interview 2: “they just buy paracetomol or some anti-malaria drug and just take it. They don’t know the gravity of the sickness, so people die almost every day of malaria. “<br><br>“We only give prophylaxis to pregnant women like the fanceda [drug], prophylaxis at first trimester, second trimester, and third trimester of pregnancy, but for malaria, no vaccines.”
Population receiving vaccines	Only children	Interview 3: “ Yeah most cases we have children are actually respond to vaccines. Adults they don’t want to receive vaccine especially when it comes to malaria.”
	Adult vaccines	Interview 2: “No, we have vaccine for adult like the hepatitis vaccines you take first, second, and third dose for adult.”<br><br>“They should take it if it is for adult if is advisable to take it.”<br><br>Interview 3: “Of course we all have the same life and we all have the same body, the same responses to sickness, the types like malaria”
Barriers	Decline vaccine	
	Personal fears/ rumors/ opinions	
	Service-related barriers	
Enablers	Vaccine acceptance	Interview 1: “Yes, they usually come for vaccination, like Fridays are our immunization day, so they come.”<br><br>“No” [to never hearing of people refuse vaccines]<br><br>Interview 2: “Not everyone but majority accepts.”

		"but people, majority come for vaccines willingly."<br><br>Interview 3: "Yeah. We have polio vaccination, you have malaria, you have other sicknesses like even recently, I heard about another vaccination for hepatitis. These are issues that we are really concerned about."<br><br>"Of course, of course. A lot of people even do ask if we have vaccine for some of these things and we are hopefully; we want to make sure we make some of these things available."<br><br>"..but I'm sure generally people do accept vaccine generally. May be except for few people but most people I believe they accept vaccine especially those that come. Most people when they come, they ask us whether we have vaccine for some of these things."
	Identified promoter factors	
Barriers	Decline MV	
	Personal fears/ rumors /opinions	Interview 2: "Some people are going with the concept that people give them vaccines that their children will not give more birth to many children because they are trying to stop birth rates. Some have those negative concepts about vaccines"
	Service-related barriers	
Enablers	MV acceptance	Interview 1: "Ya, if it can prevent from getting malaria, I will be willing,"<br><br>"Very much, I will be willing, and I will encourage everybody around my community, my people, because malaria is a killer disease."<br><br>Interview 2: "Because it is going to prevent me from getting sick and avoiding taking medicine or going to the clinic all the time."<br><br>Interview 3: "R: For other diseases? Why not? If it is a preventable mechanism? Because people em am people, I want to believe majority em except few patients

		that come for treatment in clinic may want to prevent themselves from coming for treatment; rather than am get themselves prevented by coming to clinic for treatment. So I want to believe if we have such facility, if we have em these vaccines people would love to get it with sensitization.”
	Identified promoter factors	
Barriers	Decline TBV	Interview 2: “My brother, for in fact it going to be involving adult, it’s going to be difficult because they will not accept that it’s a malaria vaccine.”
	Personal fears/ rumors /opinions	Interview 1: “They will ask if it’s really going to prevent them from getting malaria. And I think that will be the main question and also, what are the side effect.”<br><br>Interview 2: “My brother, for in fact it going to be involving adult, it’s going to be difficult because they will not accept that it’s a malaria vaccine. They have different concept that you are going to give us this that I will not get pregnant, I will not this, I will not that, that will be concept, if it’s their children, they willingly bring the kids to be vaccinated, but as long as it’s involving adult, the layman, may be you meet someone who is educated who knows about vaccine and the importance of vaccine, but for the others, even some people are educated but always have the negative concept about vaccine, so it going to be difficult.”<br><br>Interview 3: “Well questions will come like for instance, what are the advantages and disadvantages in sense you know for every action there is an equal reaction. People may want to know if it is not another problem. What the reaction if I take for instance this kind of vaccine what will be the reaction? Maybe if by giving affect you in another way or another form not only preventing you from malaria, but also the adverse effect. We want to know what are the advantages of the vaccine. I may not just want to take this vaccine, I may also want to know if this vaccine will cause harm to me n due course. That will be one of the questions that people will ask.”

	Service-related barriers	Interview 1: Well, at times, we go out of stock for malaria treatment, we will like for drugs to be always available, that is the chain of supply to be continue.<br><br>Interview 3: ": Well first of all my concern here is: if we start the vaccine we must make sure it become a reality and it is consistent because the moment you break the chain, you let people go away from that particular trend, for you to convince them to come back, it will be a problem. So we need consistency in that and we need to make sure it's available, almost always available in all health center so that emm anybody that want to be vaccinated can just step in to any to any health center of his choice to get the vaccine. But if we don't encourage government to make sure it is available almost everywhere, an at any time, it will be difficult for us to have the actual purpose for this vaccine."<br><br>"And also the other question let me don't forget would be whether is free. Sierra Leone being what it is, people may want to know the economical aspect of it whether it is free. And if it is free, how available and how affordable will it be from the other health centers because the private sector also may want to have vaccine understood. And if the private sector is supplied with this vaccine then they must do it free without cost."
Enablers	TBV acceptance	Interview 1: "Yes."<br><br>Interview 2: "R: Yes, I will be willing"<br><br>"It will improve the community and it will really help some will be willing and very happy to take and receive it."<br><br>Interview 3: "Of course……… So e mem if we have the vaccine of that nature we will be proud to give it freely to people and will encourage people to be part of the sensitization team. So that they encourage people to come and take the vaccine."
	Identified promoter factors	Interview 1: "Because it prevents me from getting malaria, I will take it"

		Interview 2: “Yes, I will be willing because it’s promoting health, if people are always sick of malaria, even now as am talking to you, there is no malaria treatment for pregnant women, but if they are being vaccinated, it will lessen the burden on us.”
Resources for health information	Sources	Interview 1_Korwama Health Center: “You know when they come to our clinic we give them health talks”<br><br>“We give talks on environmental sanitation, bed nets, all the times when they come to the clinic. So through by that, they will know malaria is transmitted and how to prevent it.”<br><br>“We give talks on environmental sanitation, bed nets, all the times when they come to the clinic.”<br><br>“Yes, before we begin the talk at times we ask them tell us what is malaria, how do you get malaria, then what would you do if you have malaria or if your child get fever at night, what would you do, we ask them all that and then they explain to us. In some cases, they tell you it’s being transmitted to them, yes at times when we ask them, they will say through mosquito bite and most of them know that.”<br><br>Interview 2_Bo Gov Hospital: “we have to talk to them, give health talks, that malaria is not transmitted by foods”<br><br>Interview 3_Egyptian Center: “We advise them to get mosquito bed or bed nets, we advise them not to stay out long at night and we advise them to always make sure they close their windows, as early as possible and their door so that they prevent malaria mosquito from coming in.”
Motivational sayings for vaccine promotion	Messages	Interview 1: "that vaccine should go along with health talks, the environment so that we don’t encourage mosquito."<br><br>Interview 2: “Yes, I will be willing because it’s promoting health, if people are always sick of malaria, even now as am talking to you, there is no malaria

		treatment for pregnant women, but if they are being vaccinated, it will lessen the burden on us."<br><br>"All about this is just to be a health talk, health educate them that this particular thing is for your own good and is not going to harm you, is going to prevent you from getting sick even if you do not have the preventative method because of their community is very filthy they have breeding site for mosquitoes. They do not sleep under mosquito net, no repellent, but you have the vaccines I will make it known that the vaccine will keep you safe from getting sick, because for now we have people that are going to expose all sort of things, but because of certain vaccines in their system, they don't get sick."<br><br>"People from the government is providing medicine for us, but the supply is not available, so if there Is a vaccine it will have less burden on us."<br><br>"It will improve the community and it will really help some will be willing and very happy to take and receive it."<br><br>Interview 3: "Before you treat somebody, you prevent somebody, I think it is better you prevent somebody than you treat the person."<br><br>Interview 3: "Of course we all have the same life and we all have the same body, the same responses to sickness, the types like malaria"
